# The Vividness of Motor Imagery Is Correlated With Corticospinal Excitability During Combined Motor Imagery and Action Observation

**DOI:** 10.3389/fnhum.2020.581652

**Published:** 2020-09-04

**Authors:** Takefumi Moriuchi, Akira Nakashima, Jiro Nakamura, Kimika Anan, Keita Nishi, Takashi Matsuo, Takashi Hasegawa, Wataru Mitsunaga, Naoki Iso, Toshio Higashi

**Affiliations:** ^1^Department of Occupational Therapy, Nagasaki University Graduate School of Biomedical Sciences, Health Sciences, Nagasaki, Japan; ^2^Department of Health Sciences, Nagasaki University Graduate School of Biomedical Sciences, Nagasaki, Japan; ^3^Department of Rehabilitation, Nagasaki Memorial Hospital, Nagasaki, Japan; ^4^Department of Oral Anatomy and Dental Anthropology, Nagasaki University Graduate School of Biomedical Sciences, Nagasaki, Japan; ^5^Department of Rehabilitation, Division of Occupational Therapy, Kumamoto Health Science University, Kumamoto, Japan; ^6^Department of Occupational Therapy, Faculty of Health Sciences, Tokyo Kasei University, Saitama, Japan

**Keywords:** motor imagery, neurophysiological assessment, corticospinal excitability, transcranial magnetic stimulation (TMS), movement imagery questionnaire-revised (MIQ-R), visual analogue scale (VAS)

## Abstract

The present study aimed to investigate the relationship between motor imagery (MI) assessment (ability and quality) and neurophysiological assessment [transcranial magnetic stimulation (TMS)-induced motor-evoked potentials (MEPs)] during combined MI and action observation (AO; MI + AO). Sixteen subjects completed an MI task playing the piano with both hands, and neurophysiological assessment was performed during the MI task. The Movement Imagery Questionnaire-Revised was adopted to evaluate MI ability, while the visual analogue scale (VAS) was adopted to evaluate MI quality. A TMS pulse was delivered during the MI task, and MEPs were subsequently recorded in the abductor pollicis brevis (APB). We found a significant positive correlation between the VAS score and the TMS-induced MEPs (*ρ* = 0.497, *p* < 0.001). These findings suggest that the VAS score could potentially reflect the corticospinal excitability during MI + AO, particularly in complex MI tasks.

## Introduction

Motor imagery (MI) is defined as a “mental simulation” or “mental rehearsal” of movement without any actual body movement (Jeannerod, 1994; Jeannerod and Decety, 1995; Decety, 1996). Prior neuroimaging studies, an activation likelihood estimation meta-analysis, and some reviews revealed that there are similar brain areas that activated both MI and actual movements, such as the premotor area (PMA), supplementary motor area (SMA), inferior parietal lobule, superior parietal lobule, cerebellum, basal ganglia, and the prefrontal cortex (Hétu et al., 2013; Hardwick et al., 2018). Moreover, prior neurophysiological studies using transcranial magnetic stimulation (TMS) revealed that the corticospinal excitability significantly increased during MI compared to rest (Kasai et al., 1997; Fadiga et al., 1999).

There have been several studies related to MI using neuroimaging technology and neurophysiological methods. In these studies, as supplementary data for accuracy of results, it is considered important to show the subject’s MI ability to form and control accurate mental images of movement and the quality and vividness of their image of the motor act (Guillot and Collet, 2005; Sharma et al., 2006). In particular, the Movement Imagery Questionnaire (MIQ; Malouin et al., 2007), Vividness of MIQ (VMIQ; Isaac et al., 1986), and Kinesthetic and Visual Imagery Questionnaire (KVIQ; Malouin et al., 2007) are used to measure the subject’s MI ability, whereas the visual analogue scale (VAS; Mateo et al., 2018) and Likert scale (Ruffino et al., 2017) describe the subjective perception of how clear and vivid the MI was. In the present study, we defined “MI ability assessment” as that which evaluates the subject’s MI ability with a task that is different from the task to be learned in MI training. We defined “MI quality assessment” as that which evaluates how vividly the MI task learned during MI training was performed.

We investigated the relationship between the neurophysiological assessment and the subjective MI questionnaire used to confirm the results in the MI study. Concerning the relationship between the VAS value as an indicator of MI quality and neurophysiological assessment using TMS, the amplitude of TMS induced-motor evoked potentials (MEPs) during MI was greater at higher VAS scores (Ohno et al., 2011; Ikeda et al., 2012). Using near-infrared spectroscopy that measures concentration changes of oxygenated hemoglobin (oxy-Hb) as a neurophysiological assessment, it was found that oxy-Hb in the SMA and PMA are similarly activated during both MI and motor execution in subjects with a VAS of 80 mm or more. Moreover, the authors suggested that it might be possible to evaluate the vividness of MI from the degree of activation of the SMA and PMA (Iso et al., 2016). Other studies also found a significant correlation between MI quality assessment using a seven-point Likert scale and neurophysiological assessment using functional magnetic resonance imaging (fMRI; Lorey et al., 2011; Zabicki et al., 2019). Other studies investigated the relationship between MI ability assessment and neurophysiological assessment using TMS induced-MEP amplitude (Williams et al., 2012) and electroencephalography (EEG; Toriyama et al., 2018) and revealed a significant correlation between MI ability assessment and neurophysiological assessment.

The MI tasks used in these previous studies were relatively simple movement tasks, such as a reach movement or a single joint movement, and almost all subjects were able to image the task vividly. In the present study, we adopted a complex and task-oriented task, as these MI tasks are not readily imaged vividly. In the past, almost all TMS studies have recorded MEPs from the muscles of the hand or upper limb. The piano task had been adopted in many TMS studies (Houdayer et al., 2016; Rossi et al., 2019). In our opinion, playing the piano requires highly complex skills, such as orderly, sequential control of individual finger movements; therefore, the piano task was suitable for the TMS study, particularly the MEPs recording from finger muscles. A previous study of the relationship between cortical motor output maps evoked by TMS and the effect of MI training adopted the piano task as an MI task (Pascual-Leone et al., 1995). The piano task could be adjusted for the level of difficulty and reflects the difference in the vividness of MI among subjects. For these reasons, the piano task was adopted as the MI task in the present study. Moreover, the piano task would be suitable to be used as a motor learning task in our next study, because it has many indicators, such as velocity, duration, and precision.

Although prior studies have investigated the relationship between neurophysiological assessment and MI ability or quality assessment, few studies have investigated the relationship among these assessments simultaneously, and there is still uncertainty about which assessment can be used as ancillary data for neurophysiological assessment to reflect greater certainty. Also, in the aforementioned study using the piano task in MI training (Pascual-Leone et al., 1995), it was revealed that motor learning progresses by MI training, but there was as much performance improvement as there was physical practice alone. Moreover, MI training led to the same plastic changes in the cortical motor output maps as those shaped by physical training. Therefore, the present study aimed to clarify the relationship between the MI ability assessment, MI quality assessment, and neurophysiological assessment using TMS. Moreover, to reveal how neurophysiological assessment and MI assessment change over time, we also analyzed the change over time due to the MI session.

## Materials and Methods

### Subjects

Sixteen healthy subjects (eight men and eight women, mean age 25.2 ± 5.0 years) were enrolled in the present study after providing written informed consent. All subjects are self-reported as right-handed.

The present study was based on the global guidelines for care in the use of TMS (Rossi et al., 2009). In the first stage of recruitment, all subjects filled out a questionnaire designed to exclude those with contraindications; however, none reported neurological impairment or contraindications to TMS. All experimental procedures were conducted following the Declaration of Helsinki. The study was approved by the local ethics committee at the Nagasaki University Graduate School of Biomedical and Health Sciences (No. 19061304).

### Experimental MI Task

**Figure 1:**
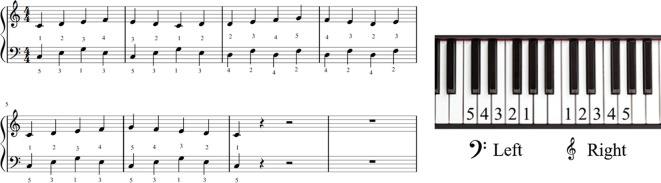
The musical score used in this experiment.

**Figure 2:**
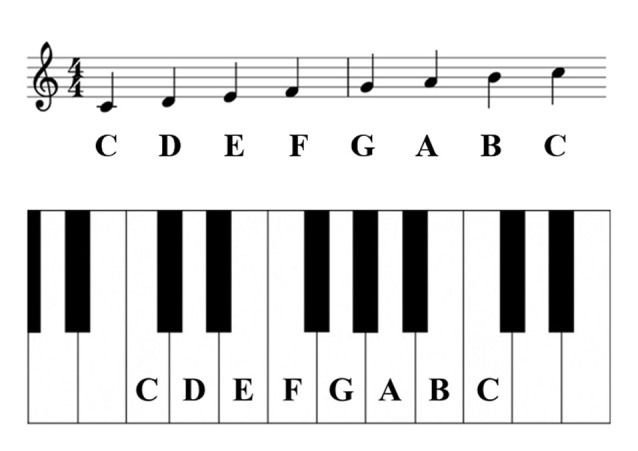
Musical notes on a piano keyboard.

**Figure 3:**
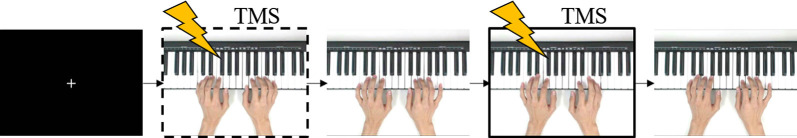
The sequence of stills from the video clip used in the motor imagery (MI) task. The frame in the dashed box is at the timing of striking a C note and the frame in the solid box is at the timing of striking a G note. During the first 150 frames, a white cross in the center of a black screen was presented. Following the action being displayed, a C note was struck at 155, 335, 635, and 880 frames (of 890 in total) after action onset (after the white cross disappears). After 506/890 frames of the action onset, a G note was struck. TMS was delivered at one of these five-time points. TMS, transcranial magnetic stimulation.

The MI task included playing the piano with both hands. The music used in the task was partially modified concerning the piano task used in a previous study (Houdayer et al., 2016), considering the difficulty of playing the piano with both hands. Subjects played the piano with a music score shown in Figure 1 (Figure 2 shows musical notes on a piano keyboard). Figure 3 shows the five frames from the video clip used in this experiment. In the present study, to trigger stimulation at a specific time and match the timing of TMS and MI, a method of practicing the motor imagery while observing the video was adopted. To create the stimulus video, a model was filmed from a first-person viewpoint playing the piano with both hands. The model has played the piano for over 10 years. The video was recorded using a web camera (c920r, Logicool; Lausanne, Switzerland) and had a duration of almost 33,000 ms (890 frames). We played the video by presenting a series of single frames, each lasting 33.3 ms (800 × 600 pixels, color depth 24 bits, frame rate 30 fps), which was sufficiently fast to produce an animation effect.

### MI Assessment

#### Neurophysiological Assessment

The corticospinal excitability was assessed in each subject by recording the MEPs induced by TMS while the subject imaged the experimental task while observing the stimulus video. To trigger stimulation at specific times, WMV files were converted to JPEG files consisting of 890 individual frames, and the stimulus video was shown in succession to obtain the animation effect. The presentation time of each frame was twice the length of the refresh interval used by the PC monitor (refresh interval = 16.67 ms).

Before the MI task, corticospinal baseline excitability at rest was assessed in each subject by recording 10 MEPs while the subject observed a white cross on a black screen under controlled conditions. The interval between the TMS stimuli was 10 s in controlled conditions. Subsequently, the experimenter instructed the subject to imagine playing the piano with both hands as if doing it for real and started to assess the corticospinal excitability during combined MI and action observation (AO; MI + AO). TMS was delivered once for each video clip, randomly at the timing of striking a C or G note. In summary, 50 trials were conducted in all MI task conditions. We used a custom-made computerized pulse-generation system. To ensure that TMS was always delivered at the correct time and that the experimental design was correctly implemented, the order of TMS delivery times (C and G notes) was randomized by using the LabView system (LabView, National Instruments; Austin, TX, USA).

Surface electromyography (EMG) activity was recorded in the right abductor pollicis brevis (APB) and the right abductor digiti minimi (ADM) muscles, using pairs of 9-mm Ag–AgCl surface cup electrodes (SDC112, GE Healthcare; Chicago, IL, USA). Surface EMG signals were amplified and filtered at a bandwidth of 5–3,000 Hz using a digital signal processor (Neuropack Sigma MEB-5504, Nihon Kohden; Tokyo, Japan). Analog outputs from a single processor were digitized at a sampling rate of 10 kHz and saved onto a computer for off-line analysis using an A/D converter (PowerLab16/30, AD Instruments; Bella Vista, NSW, Australia).

At the beginning of the experiment, we identified the optimal TMS coil position for evoking the greatest MEPs in both the right APB and the right ADM (the hot spot). TMS was delivered to the left primary motor cortex hot spot, marked with a pen on a swimming cap covering the scalp of each subject. TMS was employed *via* a 70-mm figure-eight coil connected to a magnetic stimulator (Magstim200, Magstim; Whitland, UK). The coil was placed tangentially to the scalp with its handle pointing backward and rotated approximately 45° away from the mid-sagittal line. Care was taken to maintain the same coil position relative to the scalp throughout the experiment. The resting motor threshold (MT) was defined as the lowest stimulus intensity that evoked an MEP at least 50 μV in amplitude in the right APB and ADM in 5 out of 10 trials. The test stimulus intensity was set at 110–130% of the resting MT and the size of the test stimuli ranged from 33 to 85% (mean 60.9 ± 11.9%). The mean size of the control MEP for the APB and ADM was approximately 0.5–1.0 mV. Throughout the experiments, subjects were instructed to avoid inadvertent movements that could give rise to background EMG activity. For each muscle in each trial, the 20-ms period preceding TMS triggering was checked for background EMG activity. If background EMG data was found, data from both muscles in the trial were rejected. MEP amplitude (peak-to-peak) was measured over each muscle in every trial. MEP amplitude was analyzed using peak-to-peak values and expressed as a percentage of the mean amplitude under control conditions.

#### MI Quality Assessment

The VAS has been widely used for subjective assessment of pain (Bijur et al., 2001; Suso-Martí et al., 2019) where patients mark the degree of pain on a 100-mm horizontal line. Recently, the VAS has been used for assessing the vividness of MI (Mateo et al., 2018). In this study, subjects marked a location on a 100-mm horizontal line, the two ends of which were labeled “0 = none at all” and “100 = very highly vivid image,” according to the vividness of the imagery they experienced.

#### MI Ability Assessment

All participants completed the Movement Imagery Questionnaire-Revised (MIQ-R; Hall and Martin, 1997) at the beginning of the experiment. The MIQ-R evaluates the subject’s ability to see (visual imagery) and feel (kinesthetic imagery) different movements, such as jumping, knee raising, and trunk flexion. This assessment consisted of eight separate movement items (four visual and four kinesthetic conditions). First, subjects performed the movement item, imagined the movement, and then scored their imagery using a seven-point Likert scale (1 = very hard to see/feel; 4 = neutral (not easy/not hard); 7 = very easy to see/feel, and intermediate levels). The MI ability was evaluated based on the total score; the higher the total MIQ-R score, the higher the MI ability.

### Experimental Procedure

We investigated which MI assessment (ability or quality) was strongly associated with the neurophysiological assessment.

First, to evaluate the subject’s MI ability, subjects underwent the MIQ-R. Next, to eliminate cognitive elements as much as possible in the task of playing the piano with both hands, which is an experimental MI task, we gave the subjects time to learn the order in which the keys were struck. There was no time limit, and the test was performed until the subject learned the order in which to strike. After fully understanding and confirming the MI tasks such as the timing of striking the keys by self-reporting, the neurophysiological assessment was started. TMS was performed once for each trial, for 50 stimulations during 50 trials of the MI task. The MI quality was assessed using the VAS every 10 trials to evaluate how vivid the total of 10 instances of MI tasks was imaged. In the end, MI quality was assessed five times over 50 trials. The analysis was conducted multilaterally based on the data obtained in each evaluation.

### Data and Statistical Analysis

If background EMG data was found, data from both muscles in the trial were rejected. The MEP amplitude (peak-to-peak) was measured over each muscle in every trial. MEP amplitude was analyzed using peak-to-peak values and expressed as a percentage of the mean amplitude under control conditions.

#### Confirmation of Muscle-Specific Activity During MI + AO

A previous study on MI using TMS revealed that MEPs recorded from muscles involved in the imagined movement are spatially and temporally modulated during imagined movement, as they are during actual movement (Stinear and Byblow, 2003). To confirm whether the MEPs were modulated in a muscle-specific manner during MI + AO in the present study, the data from 50 trials were statistically analyzed using two-way analysis of variance (ANOVA) comparing muscles (APB, ADM) and timing of key strikes (striking a C note with a thumb, striking a G note with a little finger). Planned *post hoc* multiple comparisons were conducted using Bonferroni’s test.

#### Transition of Neurophysiological Assessment and MI Quality Assessment Among Each Set

To check whether the MEP (neurophysiological assessment) or VAS (MI quality assessment) results changed over time, the data were statistically analyzed using ANOVA according to sessions (first, second, third, fourth, fifth). Planned *post hoc* multiple comparisons were conducted using Dunnett’s test.

#### Relationship Between Neurophysiological Assessment and MI Quality Assessment

Subjects were asked for an “MI quality assessment” (VAS) every 10 MI task trials and five VAS assessments were conducted over the 50 MI task trials. Neurophysiological assessment (TMS assessment) was conducted every MI task trial, and the average of the data obtained in every 10 trials (relative MEP amplitude) was calculated, and five MEP amplitudes were calculated in 50 trials. Spearman’s correlation analysis was performed using the corresponding data of each assessment to investigate the relationship between TMS assessment and VAS assessment.

#### Relationship Between Neurophysiological Assessment and MI Ability Assessment

Subjects were asked for an “MI ability assessment” (MIQ-R) only once before starting the MI task trial. Neurophysiological assessment (TMS assessment) was conducted every MI task trial, and the average of the data obtained in 50 trials (relative MEP amplitude) was calculated. Spearman’s correlation analysis was performed using the corresponding data of each assessment to investigate the relationship between the TMS assessment and the total MIQ-R score.

#### Relationship Between MI Quality Assessment and MI Ability Assessment

Subjects were asked for an “MI quality assessment” (VAS) every 10 MI task trials and five VAS assessments were conducted over the 50 MI task trials. On the other hand, subjects were asked for “MI ability assessment” (MIQ-R) only once before starting the MI task trial. Spearman’s correlation analysis was performed using the corresponding data of each assessment to investigate the relationship between the VAS data assessed in each session and the total MIQ-R score.

## Results

### MI Ability Assessment

The total average MIQ-R score was 47.6 ± 7.5, the total average kinesthetic score was 24.6 ± 3.6, and the total average visual score was 23.0 ± 5.1.

### Muscle-Specific Modulation of MEP Amplitudes During MI

**Figure 4:**
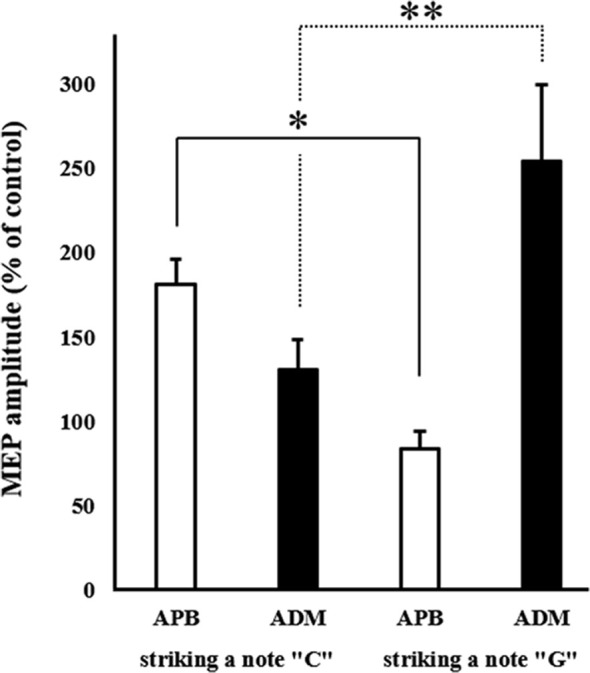
Mean MEP amplitudes over the right APB and ADM at the two different timings of TMS during combined MI and action observation (AO). Values are expressed as a percentage of control condition amplitude (*n* = 16). Data are presented as mean ± SE. The asterisk (*) and double-asterisk (**) indicate differences between conditions. *p < 0.001, **p < 0.05. ADM, abductor digiti minimi; APB, abductor pollicis brevis; MEP, motor-evoked potential; TMS, transcranial magnetic stimulation.

The mean MEP amplitudes as a percentage of control (± standard error) induced in the right APB and ADM in response to a single-pulse TMS are shown in Figure 4. Two-way ANOVA revealed a significant interaction between “Timing of TMS” and “muscle” (*F*_(1,15)_ = 17.425, *p* < 0.01).

*Post hoc* analysis revealed that MEPs recorded from the APB in the timing of “striking a C note with a thumb” significantly increased compared to the timing of “striking a G note with a little finger.” Moreover, MEPs recorded from the ADM in the timing of “striking a G note with a little finger” significantly increased compared to the timing of “striking a C note with a thumb.” From these results, the present study revealed the muscle-specific modulation of MEP amplitudes during MI + AO, in line with a previous study (Stinear and Byblow, 2003).

### Progression of MEP and VAS Scores Over Time

**Figure 5:**
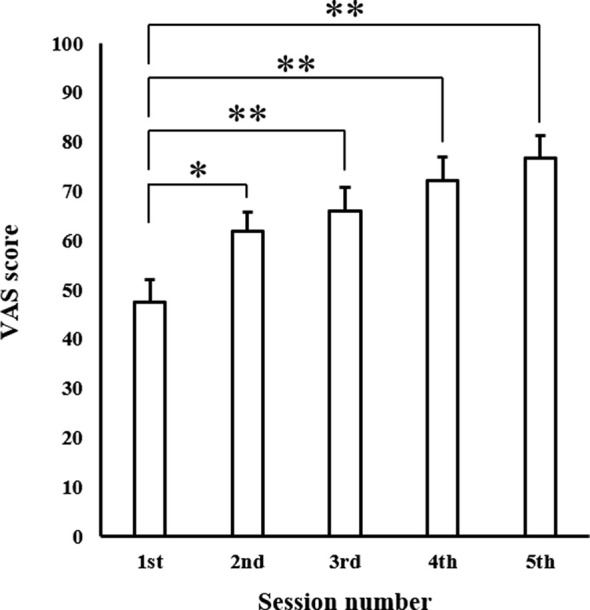
Mean MI ability assessment results in each session. Values represent VAS score (*n* = 16). Data are presented as mean ± SE. The asterisk (*) represents *p* < 0.01, and the double-asterisk (**) represents *p* < 0.001. VAS, visual analogue scale.

The mean VAS scores in each session are shown in Figure 5. One-way ANOVA revealed a significant main effect of “session” (*F*_(4,60)_ = 15.973, *p* < 0.001). *Post hoc* multiple comparisons revealed that MEPs in the second, third, fourth, and fifth sessions were significantly greater than those observed in the first session (second session: *p* < 0.01, third, fourth, and fifth sessions: *p* < 0.001).

**Figure 6:**
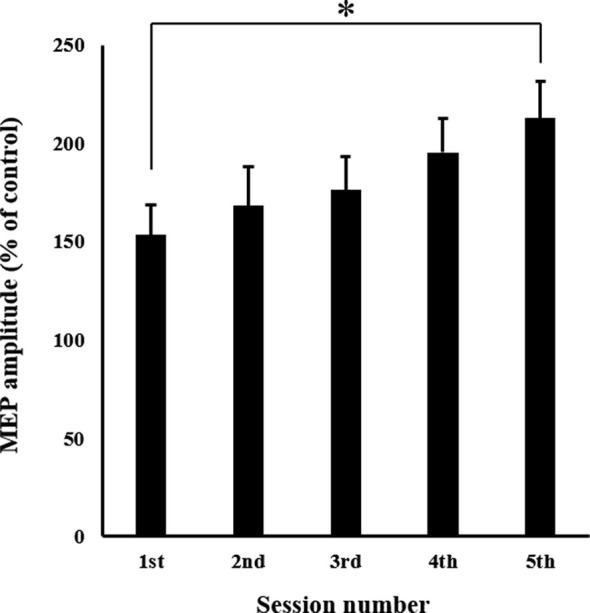
Mean MEP amplitudes over the right APB in each session. Values are expressed as a percentage of control condition amplitude (*n* = 16). Data are presented as mean ± SE. The asterisk (*) represents *p* < 0.01. APB, abductor pollicis brevis; MEP, motor evoked potential.

The mean MEP amplitudes recorded from the APB in each session are shown in Figure 6. One-way ANOVA revealed a significant main effect of “session” (*F*_(4,60)_ = 3.910, *p* < 0.01). *Post hoc* multiple comparisons revealed that MEPs in the fifth session were significantly greater than those observed in the first session (*p* < 0.01).

### Relationship Between Neurophysiological Assessment and MI Quality Assessment

**Figure 7:**
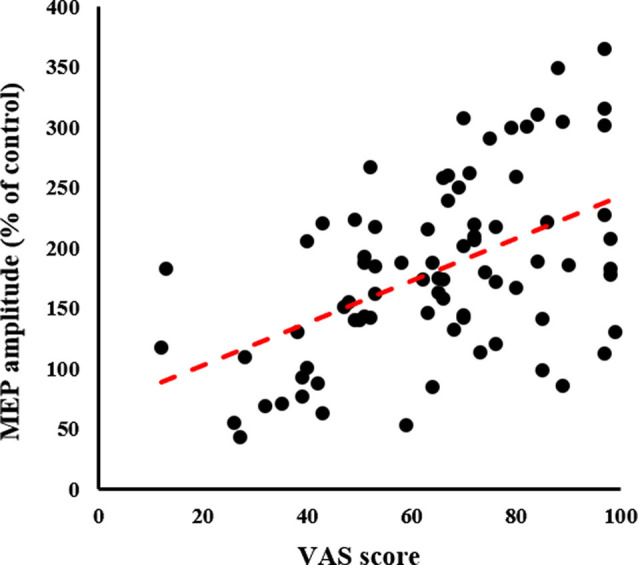
Relationship between MI quality assessment and neurophysiological assessment. The X-axis represents the VAS score and the Y-axis represents MEP amplitude as a percentage of the control condition; significant Spearman’s correlation (*ρ* = 0.465, *p* < 0.001) is noted. The higher the VAS score, the greater the MEP amplitude. MEP, motor-evoked potential; VAS, visual analogue scale.

A significant positive correlation was detected between the VAS and MEP (*ρ* = 0.497, *p* < 0.001; Figure 7).

### Relationship Between Neurophysiological Assessment and MI Ability Assessment

**Figure 8:**
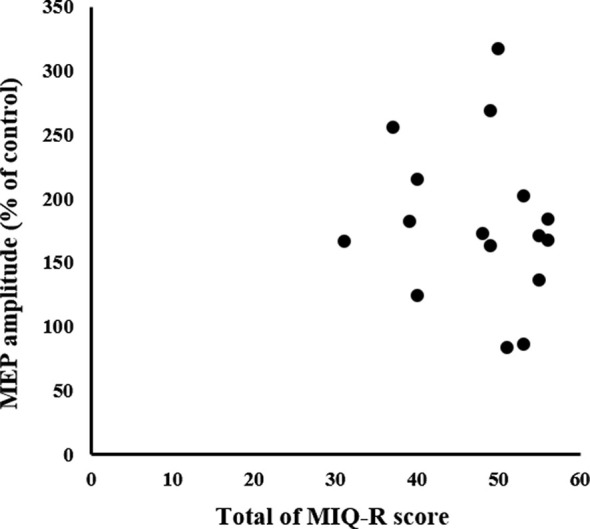
Relationship between MI ability assessment and neurophysiological assessment. The X-axis represents the total MIQ-R score and the Y-axis represents MEP amplitude as a percentage of the control condition; no correlation is seen. MEP, motor-evoked potential; MIQ-R, Movement Imagery Questionnaire-Revised.

There was no significant correlation between the MIQ-R and MEP (*non-significant*; Figure 8).

### Relationship Between MI Quality Assessment and MI Ability Assessment

There was no significant correlation between the VAS assessed in each session and the total MIQ-R score.

## Discussion

Previous studies on MI have investigated the relationship between neurophysiological assessment and MI ability assessment (Lebon et al., 2012; Wang et al., 2014; Toriyama et al., 2018) or MI quality assessment (Lorey et al., 2011). Only a few studies have investigated the multiple relationships between these assessments (Mizuguchi et al., 2019); therefore, there are still many uncertainties. To reveal which assessment reflects the outcome of the neurophysiological assessment, as supplementary data for accuracy of results, we investigated the various relationships among MI ability assessment, MI quality assessment, and neurophysiological assessment. As a result, we only found a positive correlation between the MI quality assessment, which evaluates the vividness of the tasks learned in MI training, and neurophysiological assessment. Similar to previous studies on MI using TMS, we also found that the primary motor cortex corresponding to the muscle activity activated during actual movement was activated in a muscle-specific manner. Therefore, the task itself and the accuracy of the experiment are considered valid. Moreover, we found that the neurophysiological assessment (TMS-induced MEPs) and MI quality assessment (VAS) significantly changed over time during the MI + AO session.

There are many questionnaires for MI assessments, including the KVIQ, VIMQ, Likert scale, and VAS, but we used the MIQ-R for MI ability assessment and the VAS for MI quality assessment. The former assessed the subject’s MI ability, while the latter assessed the degree of vividness for the task of imaging movement. As an analogy, we use the results of a “physical fitness test” (Henriques-Neto et al., 2020) which comprises grip strength, repeated side jumps, 50-m running, and other tests to evaluate individual motor skills. The physical fitness test is to comprehensively judge the subject’s fundamental motor ability. If this score is high, it can be interpreted that the comprehensive fundamental motor ability is high. However, when looking at individual events, it is not always possible to say that any sport can be practiced well just because the results of a physical fitness test are good. It is the same with MI ability assessment in MI, and it is considered that a person with a high MI ability score does not necessarily vividly imagine all MI tasks. In the present study, we examined the relationship between neurophysiological assessment and each MI assessment using tasks that are incompatible with the tasks used during MI ability assessment. As a result, we found a positive correlation only between neurophysiological assessment, particularly corticospinal excitability, and MI quality assessment. In a previous study, there are significant positive correlations between the MI quality assessment [i.e., perceived vividness using seven-point scale rating from very high (7) to very low (1)] and neural activation in the left ventral premotor cortex and right inferior parietal lobule by fMRI (Zabicki et al., 2019). The authors argued that the activation state of the primary motor cortex is tuned by the activation state of the premotor cortex and can, therefore, be associated with subjective vividness. Although they could not suggest a detailed mechanism, our findings suggest that the difference of vividness affected the activation of the primary motor cortex due to changes in the premotor cortex activation. In MI tasks with object-related movement, the vividness of MI is parametrically associated with neural activity within sensorimotor areas (Lorey et al., 2011). In MI tasks with finger tapping, MI quality assessment by VAS correlated with the activity of the right orbitofrontal cortex (Houdayer et al., 2016). These results support our findings and it is suggested that brain activity during MI could change depending on MI quality assessment, which is the degree of vividness in the task of MI.

In the present study, we did not find a correlation between MI ability assessment and neurophysiological assessment. In a previous study examining the relationship between MI ability assessment and neurophysiological assessment in a tennis movement task used as an MI task, tennis players and novices were evaluated for MI ability assessment, and in a neurophysiological movement task, only tennis players had a significant correlation (Fourkas et al., 2008). Similar results were found in a study on badminton players (Wang et al., 2014). However, when simple movements such as thumb opposition movements and wrist movement are used as MI tasks, a significant correlation was found between MI ability assessment and neurophysiological assessment (using TMS and EEG; Williams et al., 2012; Toriyama et al., 2018). To summarize the results of these findings, in tasks involving proficiency when experts and novices were compared, a correlation was found only for experts, whereas for simple actions that were relatively easy and could be performed by anyone, there was a correlation. Considering these previous findings, it was suggested that whether there was a correlation between MI ability assessment and neurophysiological assessment could be determined by whether the task using MI was mastered or not.

We also found that neurophysiological assessment (TMS-induced MEPs) and MI quality assessment (VAS) significantly changed over time during the MI + AO session in the present study. A previous study showed that MI training led to the development of neuroplasticity (Avanzino et al., 2015). Moreover, another study showed that corticospinal activation during MI is positively related to the magnitude of imagery-dependent motor cortical plasticity following MI training (Yoxon and Welsh, 2020). In the present study, we observed the changes over time in a short period, only 50 times, but in the future, we will assess long-term changes. It is also necessary to investigate the relationship with the performance of actual motor learning.

A limitation of this study is the small sample size; there were 16 subjects, which might not be a sufficient sample size to collect relevant data. Furthermore, we did not perform sample size estimation and power analysis before the beginning of the study. The lack of significance in some statistical tests may be due to the small sample size.

## Clinical Implication

Motor imagery training involves repeatedly performing motor imagery to improve the performance of exercise tasks, and has been applied in the fields of sports, rehabilitation, and music. Particularly, in the field of rehabilitation, motor imagery training has shown to be beneficial in the recovery of an affected upper limb and balance in some systematic reviews (García Carrasco and Aboitiz Cantalapiedra, 2016; Guerra et al., 2017). Moreover, a previous study in healthy subjects has found high vividness scores to be related to greater improvement (Ruffino et al., 2017). These findings suggest that it is important how vividly a subject can perform motor imagery in order to practice effective motor imagery training. We determined that motor imagery vividness is positively correlated with amplitudes of motor-evoked potentials, but there was no correlation between motor-evoked potentials and motor imagery ability. Our findings might be useful to evaluate how vividly a subject can perform motor imagery; however, there are many unclear points. To further explore the relationship between the effects of motor imagery training and motor imagery assessment, future studies should investigate the relationship from various aspects.

## Conclusion

MI quality assessment can be performed regardless of the type of MI task or an individual’s proficiency for the task. Therefore, MI quality assessment, which assesses the vividness of imagination, maybe a more useful assessment as supplementary data to guarantee the accuracy of results for MI studies.

## Data Availability Statement

The raw data supporting the conclusions of this article will be made available by the authors, without undue reservation.

## Ethics Statement

The studies involving human participants were reviewed and approved by Nagasaki University Graduate School of Biomedical and Health Sciences (No. 19061304). The patients/participants provided their written informed consent to participate in this study.

## Author Contributions

TMo, NI, and THi conceived and designed the experiments. TMo, AN, KA, KN, and THa performed the experiments. TMo, NI, and THi analyzed the data. TMo and JN created the experimental program. TMo, KN, NI, and THi drafted the manuscript. All authors contributed to the article and approved the submitted version.

## Conflict of Interest

The authors declare that the research was conducted in the absence of any commercial or financial relationships that could be construed as a potential conflict of interest.
